# Prokaryotic ammonium transporters: what has three decades of research revealed?

**DOI:** 10.1099/mic.0.001360

**Published:** 2023-07-14

**Authors:** Adriana Bizior, Gordon Williamson, Thomas Harris, Paul A. Hoskisson, Arnaud Javelle

**Affiliations:** ^1^​ Strathclyde Institute of Pharmacy and Biomedical Sciences, University of Strathclyde, Glasgow, G4 0RE, UK

**Keywords:** bacterial ammonium transport, transport mechanism, transporter proteins

## Abstract

The exchange of ammonium across cellular membranes is a fundamental process in all domains of life. In plants, bacteria and fungi, ammonium represents a vital source of nitrogen, which is scavenged from the external environment. In contrast, in animal cells ammonium is a cytotoxic metabolic waste product and must be excreted to prevent cell death. Transport of ammonium is facilitated by the ubiquitous Amt/Mep/Rh transporter superfamily. In addition to their function as transporters, Amt/Mep/Rh proteins play roles in a diverse array of biological processes and human physiopathology. Despite this clear physiological importance and medical relevance, the molecular mechanism of Amt/Mep/Rh proteins has remained elusive. Crystal structures of bacterial Amt/Rh proteins suggest electroneutral transport, whilst functional evidence supports an electrogenic mechanism. Here, focusing on bacterial members of the family, we summarize the structure of Amt/Rh proteins and what three decades of research tells us concerning the general mechanisms of ammonium translocation, in particular the possibility that the transport mechanism might differ in various members of the Amt/Mep/Rh superfamily.

## Introduction

The transport of ammonium (NH_4_^+^) across cell membranes is a fundamental process in all domains of life. After N_2_, ammonium is the most prevalent nitrogenous compound on earth, and for most prokaryotes, fungi and plants, it is a preferred nitrogen source [1–3]. For ammonia-oxidizing bacteria (AOB), ammonium provides energy and reducing power [4]. In contrast, for mammals, ammonium is the end product of nitrogen metabolism [2], and a key metabolite in the control of systemic acid–base balance [5, 6]. In humans, elevated concentrations of ammonium can lead to health conditions, including neurological disorders, and growth retardation in neonates and children [7]. Thus, efficient ammonium excretion is essential in detoxification of erythrocytes, kidney and liver cells [8].

For years, the prevailing view was that neutral NH_3_ species passively diffused across the membrane down its concentration gradient [9]. However, for many plants and bacteria, passive diffusion rates would be insufficient to meet cellular nitrogen requirements [10]. The pKa of the NH_4_^+^/NH_3_ equilibrium is 9.25, thus at physiological pH, 99 % of ammonium is protonated. In addition, lipid membranes were found to be impermeable to ions [11, 12], suggesting the presence of specific ammonia/ammonium (NH_3_/NH_4_^+^) transport systems.

The first evidence of the existence of ammonium translocation systems was reported while investigating amino acid transport during nitrogen starvation response in the filamentous fungi *Penicillium chrysogenum* [13]. The authors found that the activity of a non-specific amino acid permease was inhibited by ammonium, suggesting the presence of a dedicated ammonium uptake system. This was further confirmed by measuring the uptake of the ammonium analogue: ^14^C-labelled methylammonium (MeA). It was shown that MeA uptake occurred against the concentration gradient and was inhibited by ammonium, confirming the presence of a specific membrane transport system for ammonium [14].

A major advance in the field came with the molecular identification of the first bona fide genes encoding ammonium transporters, named *Mep1* in the yeast *Saccharomyces cerevisiae* [15] and *Amt1* in the plant *Arabidopsis thaliana* [16]. Two additional Mep transporters, Mep2 and Mep3, were identified and subsequently characterized in *S. cerevisiae* [17]. The first prokaryotic Amt gene was sequenced and characterized in *Corynebacterium glutamicum* [18]. Soon after, numerous Amt/Mep family members were identified and characterized in a wide range of micro-organisms, including the bacterium *Escherichia coli,* the Archeaon *Archaeoglobus fulgidus *, the yeast *Candida albicans* and the ectomycorrhizal fungi *Paxillus involutus* and *Hebeloma cylindrosporum* [19–24].

In 1997, based on a sequence similarity of 20–25 % between the human red blood cell rhesus protein (RhAG) and existing Amt/Mep family members, the Rh proteins were identified as potential mammalian ammonium transporters [25, 26]. Soon afterwards, RhAG and RhGK (now called RhCG), a homologue expressed in human kidney cells, were shown to facilitate ammonium uptake in a *S. cerevisiae* triple-*mepΔ* strain, deprived of its three endogenous Mep ammonium transporters. This provided the first functional evidence that Rh proteins are able to translocate ammonium [27]. In parallel, the ammonium transport activity of RhBG, a third member of the Rh50 glycoprotein group, was characterized in human and mouse nonerythroid tissues [28, 29]. Rh homologues were later identified in many lower organisms [30, 31], including the green alga *Chlamydomonas reinhardtii*, the worm *Caenorhabditis elegans* and the nitrifying bacterium *Nitrosomonas europaea* [32–34].

The physiological relevance of Amt/Mep/Rh proteins extends beyond their role in ammonium acquisition as a nitrogen source. In the baker yeast *S. cerevisiae* and the human pathogen *C. albicans*, for instance, the Mep2 protein was found to act as a sensor required for the initiation of filamentous growth [35]. This dimorphic change is an essential process in the virulence of *C. albicans* [36, 37]. The function of Rh proteins was first revealed using a mouse knockout model; in renal excretion of ammonium, Rhcg absence causes a deregulation of blood pH, leading to lethality according to the intensity of the applied acid load [38]. This phenotype in mouse knockouts of Rhcg is reminiscent of a human syndrome called distal renal tubular acidosis (dRTA) [39]; however, so far, no relation between RhCG malfunction and dRTA has been shown.

Correlations between Rh proteins mutation/dysfunction and human pathologies have also been documented. In red blood cells (RBCs), RhAG mutations are associated with Rh deficiency syndrome (Rh_null or_ Rh_nod_), characterized by the lack of Rh antigens on RBCs [40, 41]. Overhydrated hereditary stomatocytosis (OHSt) is a rare dominantly inherited haemolytic anaemia, characterized by leakage of important monovalent cations (K^+^, Na^+^) through malfunctioning RhAG [42, 43]. In addition, the *rhbg* and *rhcg* genes have been proposed to act as potential tumour suppressors in human oesophageal squamous epithelial cancers [44] and mouse brain tumours [45]. Finally, *rhcg* has been identified as a candidate gene for early-onset major depressive disorder [46].

Many functional studies aimed at elucidating the mechanism of transport by the bacterial Amt/Rh proteins lead to a considerable controversy regarding the substrate and mechanism of transport [47]. Hence in this review, concentrating on bacterial transporters, we will present the structure of Amt/Rh proteins and discuss in detail our current knowledge of the mechanisms of ammonium translocation in prokaryotes.

## Mechanism of ammonium transport

Various mechanisms were proposed before the structure of ammonium transporters was determined in 2004. The transport mechanisms proposed before 2004 are summarized in Table 1, followed by a detailed discussion of the structure of bacterial Amt/Rh and, finally, an exploration of whether our expectations of solving the mechanism of transport based on these structures has been met.

**Table 1.** Reported mechanisms in Amt/Mep/Rh before 2004

Organisms	Proteins	Systems	Methods	Mechanisms	References
*L. esculentum*	Amt1;1	Oocyte	EP	Δψ-driven NH_4_^+^ uniporter	[53, 54]
	Amt1;2	Oocyte	EP	Δψ-driven NH_4_^+^ uniporter	[54]
* C. glutamicum *	AmtB	* C. glutamicum* cells	TA	Δψ-driven NH_4_^+^ uniporter	[52]
*H. sapiens*	RhAG	Oocyte	TA	Electroneutral NH_4_^+^/H^+^ antiporter	[58]
		Red blood cells	TA	NH_4_^+^ exporter	[57]
	RhBG	Oocyte	EP/TA/pH	Electroneutral NH_4_^+^/H^+^ antiporter	[55]
	RhCG	Oocyte	EP/pH	Transport of NH_4_^+^ and NH_3_	[56]
*S. cerevisiae*	Mep1-3	Yeast	TA	NH_3_ channel	[51]
* S. typhimurium *	AmtB	* S. typhimurium* cells	GE	NH_3_ channel	[89]
* E. coli *	AmtB	* E. coli* cells	TA/GE	NH_3_ channel	[49]

EP, electrophysiology; TA, ^14^[C]-methylammonium transport assay; pH, pH measurement; GE, growth experiment.

In 1985, the prevailing view was that these systems function in active transport of NH_4_^+^ to concentrate it within the cell [48]. A decade later, Kleiner’s ruminations could be tested following the initial identification of genes encoding *bona fide* ammonium transporters [15, 16]. Rather than providing elucidation of the mechanism, however, the lines of investigation quickly gave rise to a variety of differing perspectives and conclusions.

Some of the earliest characterization of Amt/Mep/Rh-mediated transport and accumulation of [^14^C]methylammonium (MeA), an ammonium analogue, supported proposals that the family were capable of energy-dependent concentration of the ammonium ion, NH_4_^+^ [16, 17].

This view would be challenged by work from Sydney Kustu’s laboratory who generated an *E. coli* strain with a disrupted *amtB*. The resulting mutant matched wild-type (WT) growth at pH 7 across a range of ammonium concentrations, but was slower at pH 5 under ammonium limitation (<1 mM NH_4_^+^). The pKa of ammonium is 9.25, so the relative concentration of the uncharged species decreases at acidic pH, and this was taken as evidence that AmtB recognizes NH_3_ [49]. In addition, they reported that *Salmonella typhimurium ‘*metabolically trapped’ NH_3_: metabolizing it immediately rather than accumulating it in the cytoplasm. Follow-up work revealed that, when grown on alternative N-sources, *S. typhimurium* can use AmtB to excrete intracellular ammonia [49, 50]. Taken together, the authors argued that AmtB was a passive channel that merely enhanced the pre-existing rate of NH_3_ diffusion. The channel hypothesis was extended to eucarya, with reports that NH_3_ was the natural substrate of the MEP proteins of *S. cerevisiae*. Here, Soupene *et al*. explained previous observations of MeA accumulation as the result of energy-dependent sequestration of MeA into acidic vacuoles. This was evidenced by a significant decrease in MeA accumulation in yeast strains deficient in vacuolar H^+^-ATPase, which is responsible for the acidification of said compartments [51]. Within the discussion, the authors speculate that Rh proteins might also be channels, but for CO_2_ rather than NH_3_.

Taken in isolation, these studies suggest a growing case for Amt/Mep/Rh proteins as simple channels. However, contemporaneous studies were also providing support for a more involved transporter-like role for the Amt/Mep/Rh family.

In 2001, Meier-Wagner *et al*. characterized AmtB from *C. glutamicum* using [^14^C]MeA uptake assays [52]. They showed that AmtB-mediated methylammonium uptake was dependent on membrane potential and reported that the Km for methylammonium was unaffected by a pH shift of 2.5. This supported the conclusion that methylammonium (and by extension ammonium) was the substrate, not methylamine/ammonia [52]. Ludewig *et al*. provided more direct evidence for active transport in plant Amt proteins. Specifically, they expressed AMT1;1 and AMT1;2 from *Lycopersicon esculentum* in *Xenopus laevis* oocytes and measured charge translocation in the presence of ammonium via a voltage clamp [53, 54]. The currents were dependent on both voltage and NH_4_^+^concentration, and unaffected by changes in pH. From this, the authors concluded that these proteins were NH_4_^+^ uniporters.

*Xenopus* oocytes were subsequently used to probe the mechanism of human RhBG and RhCG. RhBG was reported to be electroneutral, with the authors concluding that it was likely an NH_4_^+^/H^+^ antiporter [55]. In contrast, an NH_4_^+^-dependent current was observed for human RhCG, supporting a similar NH_4_^+^ uniport mechanism to *L. esculentum* AMTs. The authors observed that NH_3_ concentration altered the affinity of RhCG, hinting at a more complex mechanism involving transport of both species [56]. Finally, mixed results were obtained for RhAG. Transport assays carried out on RhAG-expressing oocytes supported NH_4_^+^/H^+^ antiporter activity, while a comparable assay in RBCs suggested that RhAG had the capacity to export NH_4_^+^ [57, 58].

As portrayed by these studies, the early 2000s featured a confluence of disparate hypotheses pulling the field in different directions (Table 1). The controversy was the result of the lack of quantitative kinetic data characterizing the activity of Amt/Mep/Rh proteins at the single channel level. Essentially all functional studies were carried out using intact cells, cell-derived vesicles or substrate analogues. In all of these systems, the parameters measured to analyse substrate conduction (pH change, electric current, uptake of labelled analogue) are likely to be affected by other physiological phenomenon, requiring careful controls [47].

Hence from the contemporary literature, it was difficult to confidently proclaim that Amt/Mep/Rh were channels or transporters, or even that a single mechanism was conserved across the family. Thus, it was hoped that the eventual purification of active protein and solving of crystal structures would provide clarity and elucidate the truth of the mechanism.

## Structural characterization

### Membrane topology and secondary structure

The topological organization of Amt proteins was first determined *in vivo* for the ammonium transporter AmtB from *E. coli* using alkaline phosphatase (PhoA) and β–galactosidase (LacZ) reporter fusion proteins [59]. The results supported an 11 TM helices (TMH) model with the N-terminus facing the periplasm and the C-terminus located in the cytoplasm [59]. Prior sequence analysis had predicted a 12th helix [24], but these results suggested that the first theoretical helix was a signal peptide that would be cleaved following correct folding or insertion of the protein into the membrane [59]. The high-resolution structure (see paragraph below) demonstrated that the 11 TMH model and general topology observed in AmtB is conserved across Amt/Mep proteins; human rhesus proteins, however, have been shown to possess 12 TM helices, with both the N-terminus and C-terminus located in the cytoplasm [60].

### Tertiary and quaternary structure

Investigation into the oligomeric state of Amt/Mep/Rh was prompted by the observation of crosstalk between the different Mep isoforms in *S. cerevisiae*, where expression of an inactive Mep1 inhibited the activity of Mep2 and Mep3 [61]. Subsequently the *E. coli* AmtB, the first Amt/Mep protein to be purified, was subjected to a combination of analytical ultracentrifugation (AUC) and size-exclusion chromatography (SEC). Through these techniques it was determined that, when solubilized in dodecyl-β-maltopyranoside (DDM), AmtB purifies as a homotrimer with a molecular mass of ~140 kDa [62]. This does not correspond to the expected mass of the trimer (~130 kDa), likely due to partial unfolding of hydrophobic domains following SDS binding [63]. In 2004, the first ordered 2D crystals of AmtB were obtained, with cryo-electron microscopy, generating a low resolution (12 Å) projection map, and atomic force microscopy was used to obtain higher resolution topographs [64]. Both methods supported the trimeric structure of AmtB and suggested that each monomer exhibited pseudo-twofold symmetry

These results validated that the first high-resolution crystal structures of AmtB were solved [65, 66]. The general structure of AmtB was also later characterized using a combination of small-angle X-ray scattering (SAXS) and small-angle neutron scattering (SANS) to obtain detailed structural information on AmtB stabilized in the detergent DDM [67]. These structures confirmed the trimeric organization of AmtB and provided an unprecedented insight into the structure of the translocation pathway, enabling investigation into the mechanism of ammonium transport. Over the last two decades, high-resolution crystal structures have been solved across the Amt/Mep/Rh family, including rhesus proteins from *N. europaea* and *Homo sapiens*, fungal Mep2 from *S. cervisae* and *C. albicans*; and Amt from *Archeaoglobus fulgitus* and *Candidatus Kuenenia stuttgartiensis* [60, 68–71].

As AmtB is the archetypal member of the family, with over 20 high-resolution structures in the Protein Databank (PDB), its structure will be discussed in detail, followed by a series of comparisons to other solved structures of bacterial Amt/Rh proteins.

### Structure of *EcAmtB*

**Fig. 1.**
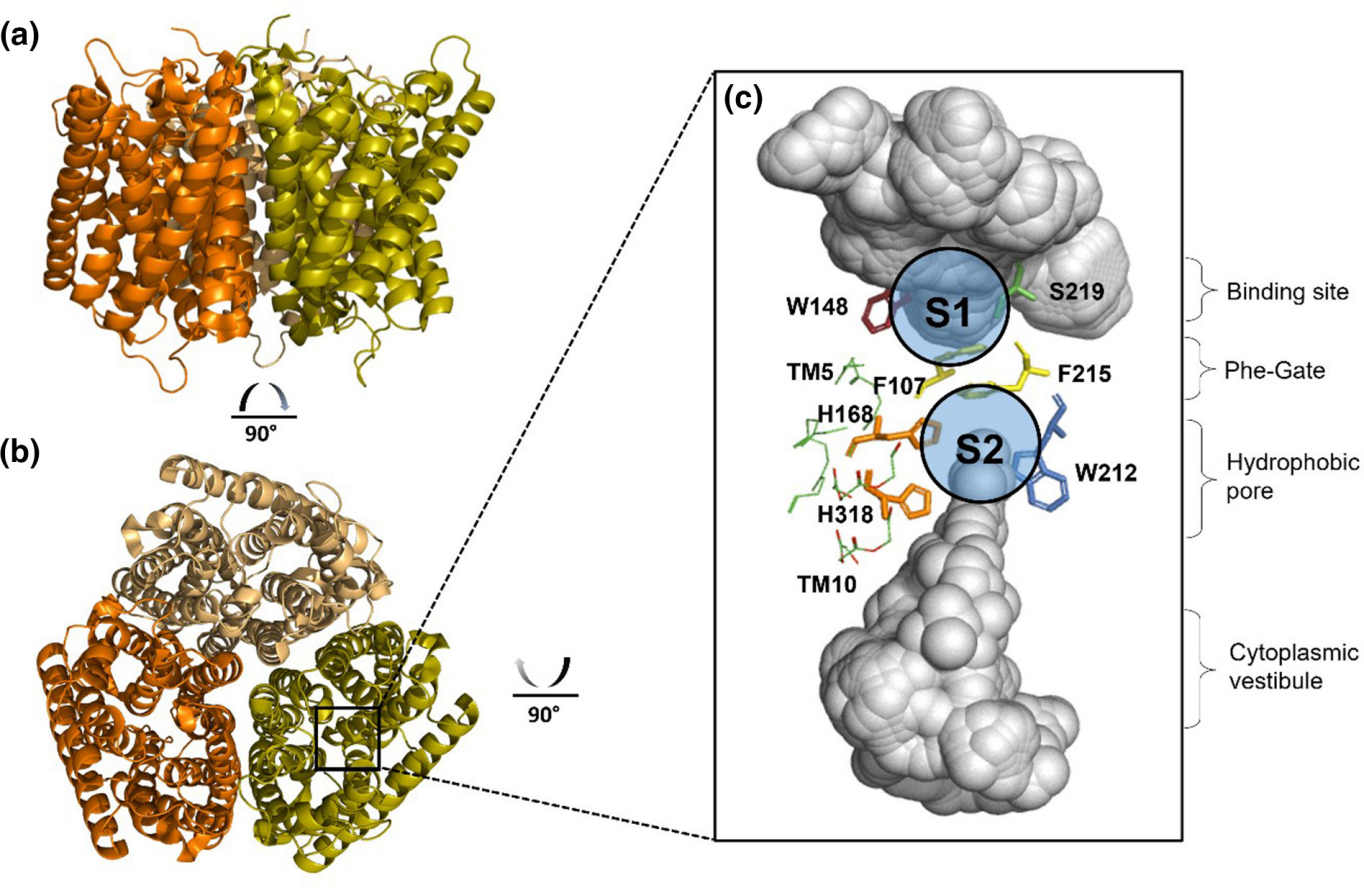
Structure of AmtB from *E. coli *. (**a**) View of the AmtB trimer from the side, and (b) from the top, with each monomer represented in olive, orange, or yellow. (**c**) Expanded view of the pore region of a single monomer with the water-accessible volume represented in grey. Highly conserved residues are shown in ball and stick representation for the ammonium-binding sites S1 and S2 (blue circle), phenylalanine gate (yellow) and central pore (grey), with displayed parts of the two transmembrane helices TM5 (His168) and TM10 (His318).

Consistent with predictions [62, 64], AmtB crystalized as a homotrimer, with each monomer conforming to the 11 TMH model (Fig. 1) [59, 65, 66]. Crucially, these high-resolution structures led to the indentifcation and characterization of the potential substrate translocation pathway located in the centre of each monomer and the identification of four key features of mechanistic interest within the translocation pathway (Fig. 1):

1. An NH_4_^+^-binding site within a vestibule in the periplasmic face of the protein named S1 site.
2. A constriction imposed by two stacked phenyalanine residues that separates the S1 site from the hydrophobic pore, named the ‘Phe-gate’.
3. A second potential binding site named S2 followed by a narrow pore lined with hydrophobic residues and containing the characteristic ‘twin-His’ motif of the family.
4. A cytoplasmic vestibule.

### S1-binding site

The high-resolution structure revealed a putative NH_4_^+^-binding site nestled at the bottom of a periplasmic vestibule in each monomer [65, 66]. As AmtB is only expressed under ammonium-limited conditions, the S1-binding site would be necessary to ensure a high affinity for the substrate and confer efficient scavenging for ammonium at low concentration in the surrounding environment [72]. The binding site (S1) is delineated by the residues S219, W148 and F107 (Fig. 1). At this site, NH_4_^+^ could be stabilized via π-cation interactions with the phenyl rings of W148 and F107 alongside hydrogen bonding with S219 [65, 66]. Javelle *et al.* demonstrated that thallium (which has a similar size and coordination to ammonium) can bind at this position, and that thallium competitively inhibits MeA uptake, strongly supporting that the S1 is capable of binding NH_4_^+^ [73].

**Fig. 2.**
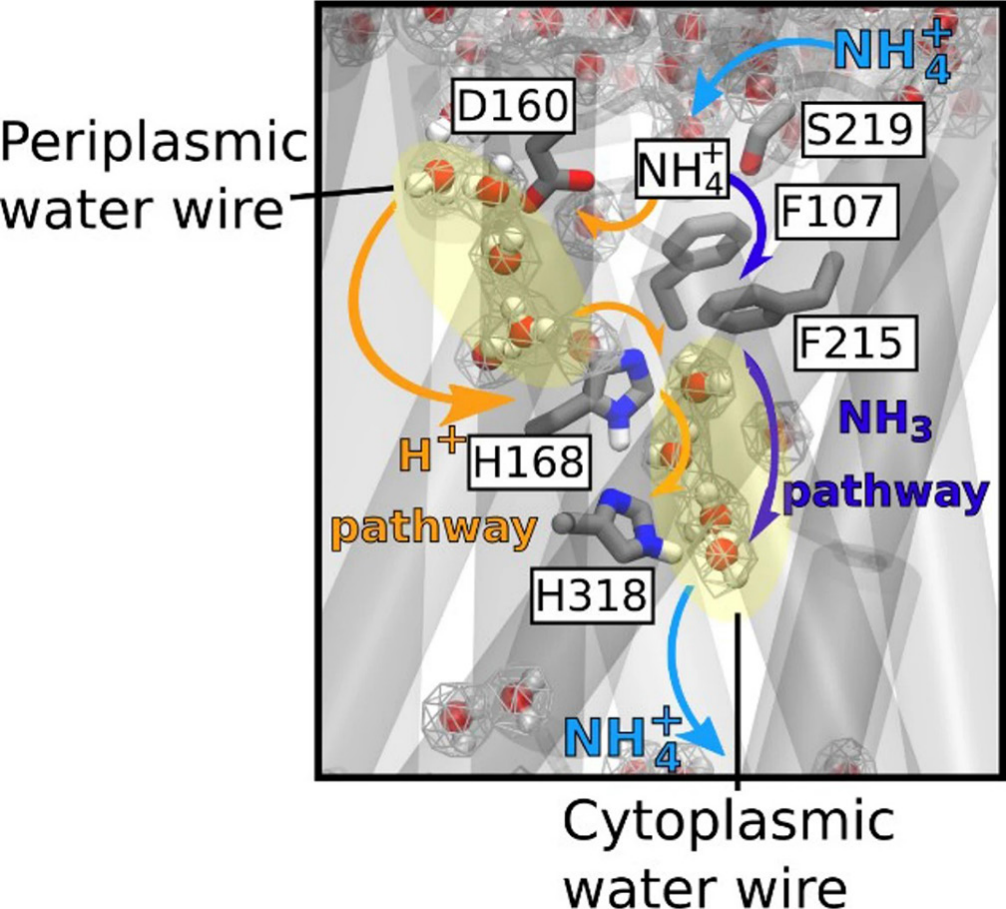
Mechanism of electrogenic NH_4_^+^ translocation in AmtB. NH_4_^+^ is deprotonated at the periplasmic face of the protein, allowing passage of uncharged NH_3_ molecules through the central part of the hydrophobic channel (depicted by dark blue arrow). A parallel passage of H^+^ into the cytoplasm is enabled by the polar conduction route formed by two internal water wires (WWs) that span across the membrane H^+^ (depicted by orange arrows). NH_3_ re-protonation likely occurs near the cytoplasmic exit. Reproduced from [76].

D160 lies in the proximity of the S1 site (Fig. 2) and is highly conserved, implying a functional or structural role [59, 74]. However, the crystal structures rule out a role in binding, as the carboxy function of D160 is buried too deeply in the pore to directly create an electrostatic interaction with NH_4_^+^ [65].

### Phe-gate

The second key structural feature of AmtB is the Phe-ate. Positioned immediately after the S1-binding site, the stacked phenyl rings of F107 and F215 form a constriction that completely blocks access to the pore from the S1 site (Fig. 1) [65]. The arrangement of F107 and F215 was identical in all the AmtB crystal structures (including AmtB co-crystalized in the presence of ammonium or different variants), but the authors suggested that the rings would have to be dynamic, as they completely block the translocation pathway [73, 75].

### Hydrophobic pore and twin-His motif

Immediately after the Phe-gate, a potential second binding site, named S2, mirrors the S1 site present in the periplasmic vestibule (Fig. 1) [65, 66, 73]. This potential binding site is delineated by the residues F215, W212 and H168. At this position, NH_4_^+^ may be stabilized via π-cation interactions with the phenyl rings of W212 and F215 alongside hydrogen bonding with H168 and a water molecule in the pore. The S2 site is followed by a narrow and strongly hydrophobic pore that leads to the cytoplasm. The hydrophobic nature of the pore represents a highly energetic barrier against the movement of ions [66]. Two highly conserved histidine residues, H168 and H318, protrude into the lumen of this pore, forming the family’s characteristic twin-His motif [75]. Whilst the hydrophobic residues in the pore can be variable, the twin-His motif is highly conserved, indicating a functionally important role [66, 75].

Remarkably, a series of four residual electron densities within the hydrophobic pore were recorded only after soaking the crystal in ammonium sulfate; indicating that they were ammonium molecules [65]. In contrast, another study observed these densities regardless of the presence or absence of ammonium [66]. Since at 1.35 Å molecules of H_2_O and NH_3_ are not readily distinguished, it was hypothesized that these densities are equally likely to be water molecules [66]. These densities would later form the foundation of different hypotheses in the mechanism (see below [76, 77].

### Cytoplasmic vestibule

The translocation pathway terminates at a vestibule in the cytoplasmic face of the protein. Unlike the periplasmic vestibule, no discernible binding site was observed in the structure. In addition, there was no barrier between the vestibule and the pore, resulting in a clear asymmetry between the periplasmic and cytoplasmic ends of the channel. Interestingly, AmtB was crystallized in two different space groups, with the cytoplasmic vestibule in different conformational states in each crystal. In one conformational state, the residues around the N-terminus of TM10 are positioned differently, such that they obstruct the cytoplasmic exit and create a purely hydrophobic environment [66]. In the other conformational state, the residues do not obstruct the pore exit and result in a more polar ‘open’ configuration [66]. This evidence of conformational change within the vestibule suggested a functionally relevant role – which is still unknown. A later extensive site-directed mutagenesis study of the *E. coli* AmtB transporter revealed that the C-terminal region might mediate co-operativity between the three subunits of the protein, indicating conformational changes in the C-terminal tail and potentially the cytoplasmic vestibule [78]

### Additional Amt/Rh structures

#### AmtB in complex with GlnK

In *E. coli *, the activity of AmtB is regulated via a physical interaction with the PII protein GlnK [74, 79, 80]. The PII protein family, the most important family of signal transduction proteins in prokaryotes, forms homotrimers with a compact barrel-like shape. A flexible loop, named the ‘T-loop’, protrudes from the upper face of the barrel. The X-ray structure of the GlnK–AmtB complex has been simultaneously resolved by two different groups [60, 81]. GlnK and AmtB form a GlnK3 : AmtB3 complex at a 1 : 1 ratio. Interestingly, the C-terminal tail of AmtB and the T-loop of GlnK are structurally defined in the complex due to the stabilizing interaction between the two proteins [60, 81], whereas they are not in the individual crystal structures [65, 66]. The T-loop of GlnK in the complex is composed of two anti-parallel β-strands and this conformation allows a perfect fitting of the loop into the cytoplasmic vestibule of AmtB, blocking its activity.

#### *A. fulgidus* Amt1

The crystal structure of *Af*Amt1, an ammonium transporter from the Archeaon *A. fulgidus *, was first solved in 2005 [68]. The general topology of *Af*Amt1 was highly similar to that of AmtB, following the same 11 TM model – with only the signal sequence missing. In addition, *Af*Amt1 retains the pseudo-twofold symmetry seen in AmtB, loosely mirroring TM1-5 and TM6-10 [65, 68].

**Fig. 3.**
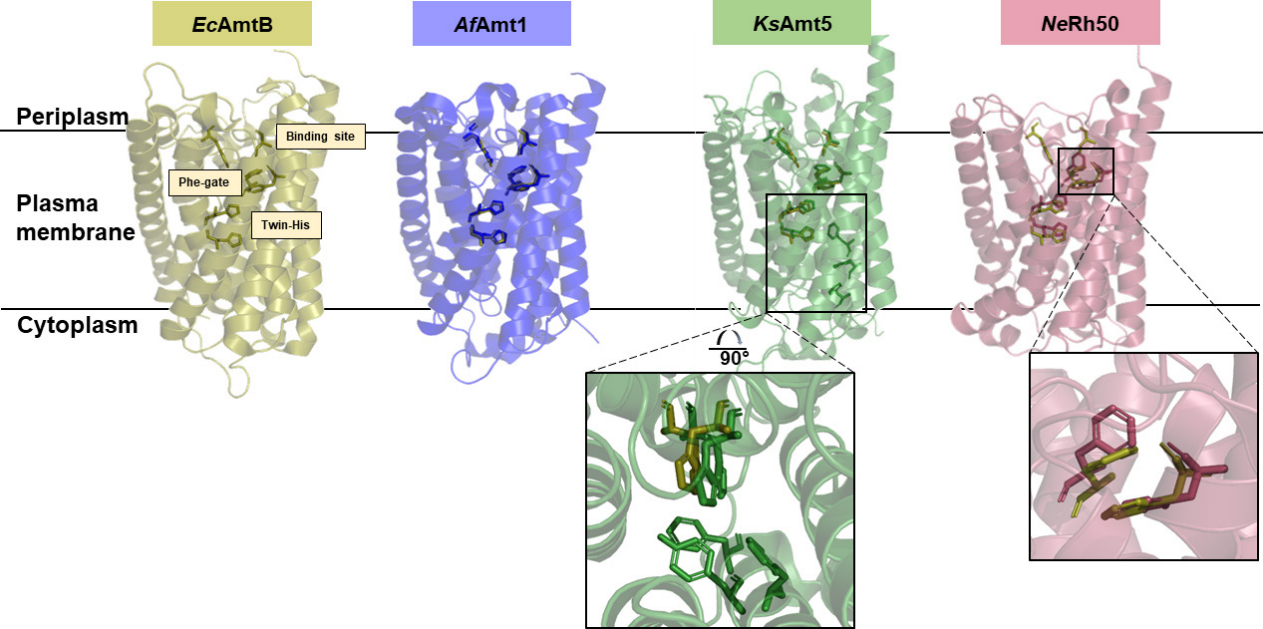
Comparison of the translocation pathway of ammonium transporters present in bacterial representatives. Single monomer of AmtB from *E. coli* (yellow), Amt1 from *A. fulgitus* (blue), *Ks*Amt5 from *K. stuttgartiensis* (green) and Rh50 from *N. europaea* (red). Crucial residues forming the binding site, phenylalanine gate and twin histidine motif from AmtB are overlaid with corresponding residues in the rest of bacterial representatives for comparison. Most critical differences are highlighted by boxes.

Similarly to the S1 binding site identified in AmtB, *Af*Amt1 also possessed a serine (S208) and tryptophan (W137), which could stabilize a molecule of NH_4_^+^ in the vestibule (Fig. 3). Whilst Andrade *et al*. [68] observed a strong electron density in this region, they were unable to correlate this density with the presence or absence of ammonium. The paired phenylalanine residues beneath the periplasmic vestibule were conserved in *Af*Amt1 and, as with AmtB, were positioned such that their rings occluded access to the channel. Interestingly, Andrade *et al.* observed higher flexibility in the sidechain of F204, leading them to conclude that the residues move during the translocation event. While Khademi *et al.* [65] observed several density peaks within the hydrophobic pore after soaking the crystal in ammonium sulfate and Zheng *et al*. [66] observed the densities in both the absence and presence of ammonium, Andrade *et al*. [68] did not observe densities in either condition.

#### *Ca.* Kuenenia stuttgartiensis Amt5

The solving of the crystal structure of *Ks*Amt5 from the anaerobic ammonium-oxidizing bacterium *Ca*. *Kuenenia stuttgartiensis* revealed that the general topology of *Ks*Amt5 is similar to that previously described for AmtB and *Af*Amt1, although the structure differs in key ways [70].

Firstly, while the binding site (W144/S227), the Phe-gate (F103/F223), and the twin-His motif (H171/H326) are all conserved, the crystal structure revealed a slight shift (~ 0.8 Å) of the twin-His motif into the channel. Moreover, three additional bulkier side chains (F27, Y30, F34), non-conserved in other Amts proteins, were identified in the translocation pore (Fig. 3) [70]. This arrangement seems to occlude the translocation pore (Fig. 3, box), potentially preventing NH_4_^+^ translocation. Indeed, using solid supported membrane electrophysiology (SSME) [82], Pflüger *et al.* demonstrated that *Ks*Amt5 binds ammonium but does not translocate it across the membrane [70]. In the structure, the second histidine (H326) residue is linked via H-bonding across two putative water molecules to an unprecedented NH_4_^+^-binding site. At this position two tightly coordinated ammonium cations have been identified. Unlike in other proteins, it is positioned within the membrane and could represent a selective high-affinity NH_4_^+^-binding site. The authors suggested that in *Ks*Amt5 the substrate binds onto the high-affinity binding sites, leading to structural rearrangements that trigger signalling [70]. These marked structural and functional differences imply a distinct role for this Amt.

Compared to *AmtB* and *Af*Amt1, *Ks*Amt5 features an extended C-terminal tail with high similarity to the histidine kinase (HK) domain observed in bacterial two-component systems [70]. Although the overall crystal structure obtained was high resolution, the HK domain was highly disordered due to its relative flexibility and thus remained undefined. Despite this, Pflüger *et al.* demonstrated HK-mediated phosphorylation to be ammonium concentration-dependent, with a significant increase under nitrogen limitation conditions (5–10 mM) [70].

Thus, *Ks*Amt5 represents a system wherein an Amt protein has been repurposed as a specific ammonium receptor modulating signal transduction to its HK in response to NH_4_^+^ concentration. This unusual arrangement may have evolved to complement the unique physiology of anammox bacteria, where ammonium catabolism is highly dependent on the ammonium concentration within its unique organelle, the anammoxosome. Whilst *Ks*Amt5 is the first to be characterized, other naturally occurring sequences combining Amt with other protein domains have been observed [83]. It is possible that similar examples will be observed in other bacteria.

#### *N. europaea* Rh50

In 2007 the first high-resolution (1.3 Å) structures of a rhesus protein from the chemolitotrophic ammonium-oxidizing obligate bacterium *N. europaea* were published [69, 84]. This was a milestone moment, as it not only allowed for structural insight into Rh50 itself, but also enabled comparison to the structures of *AmtB* [65, 66], *Af*Amt1 [68] and *Ks*Amt5 [70].

Previous *in silico* work predicted that Amt and Rh had 11 and 12 TM helices, respectively [24]. However, the crystal structure of *Ne*Rh50 revealed only 11 TM helices, which Lupo *et al.* hypothesized was due to TM helix 12 being cleaved off when expressed in *E. coli* [69].

Of the key residues seen in the AmtB S1-binding site (F107, W148, S219), only the phenylalanine is conserved in *Ne*Rh50 (Fig. 3). However, the vestibule structure is not conserved, and no ammonium-binding site is seen in *Ne*Rh50. The Phe-gate is conserved but altered in comparison to that seen in AmtB (Fig. 3). F110 is tilted in *Ne*Rh50, creating an opening for a water molecule 2 Å deeper in the pore. A similar pocket is present directly above the first histidine of the twin-His motif and contains two water molecules [69, 84]. Beyond this change, the twin-His motif is conserved and similar in both Rh and Amt.

Interestingly, while residual electron densities have been seen in the hydrophobic pore of AmtB, they were not observed here [66, 69]. The cytoplasmic vestibules of AmtB and Rh50 are very similar, but the asymmetry between the periplasmic and cytoplasmic vestibules in Rh50 is less pronounced due to the lack of the S1-binding site (Fig. 3).

In contrast to prior speculation, based on immunoprecipitation analysis of the erythrocyte Rh complex [85], that the human rhesus proteins would form heterotetramers [85], Lupo *et al.* concluded that they are certainly trimeric [69]. This was based on the trimeric structures of *Ec*AmtB and *Ne*Rh50, and comparative analysis of their respective monomer interface regions. Soon afterwards, the crystal structure of mammalian RhCG was resolved by Gruswitz *et al.* [60]. The protein was proved to retain the trimeric organization and the pseudo-twofold symmetry between TMH1-5 and TMH6-10 observed in Amts [60], confirming previous assumptions [69].

## Did the protein structures solve the transport mechanism mystery?

Despite the wealth of structural information, the mechanism of ammonium transport has remained elusive for 10 years following the publication of the structure of AmtB. All the structures show a very similar conformation, reflecting the inward-facing state of the protein, irrespective of the presence or absence of ammonium. Moreover, the hydrophobicity of the pore acts as an energetic barrier for ion translocation and this structure is highly conserved across the family [60, 65, 68, 69, 71]. This apparent structural inflexibility, combined with the hydrophobicity of the pore, lead to the initial conclusion that the Amt/Mep/Rh family were electroneutral NH_3_ channels, rather than active transporters, which generally involve large conformational changes during the translocation cycle [86]. This assumption was supported by *in vivo* and *in vitro* experiments. Khademi *et al.* adapted a fluorescence-based assay to measure the influx of ammonia into proteoliposomes containing AmtB by monitoring the pH-sensitive fluorescence of 5-carboxy fluorescein (CF). Following mixing of CF-loaded proteoliposomes with NH_4_Cl, the internal pH rose, reflecting influx of NH_3_ (Table 1) [65]. However, these results have never been reproduced despite extensive effort, questioning the validity of the initial conclusion [47].

Javelle *et al.* developed an ‘unwashed’ MeA transport assay (for technical details of the unwashed assay, see [80]). These data report that strains expressing AmtB show a complete linear dependence over a range of external MeA concentrations, indicating a passive diffusion of the substrate down an electrochemical gradient, in agreement with a channel-like activity rather than transporter-like activity (Table 2 [80]). The same technique was used to assess the mechanism of Rh50 transport, with a similar result to the that obtained for AmtB, leading the authors to conclude that Rh50 from *N. europaea* also acted as a channel rather than a transporter [87]. In addition, the restoration of ammonium-dependent growth to a yeast *Δmep* mutant by Rh50 was more effective at higher pH values, providing further evidence in support of Rh50 translocating NH_3_ rather than NH_4_^+^ (Table 2 [87, 88]).

In parallel to NH_3_ transport, Rh proteins were proposed to facilitate CO_2_ across the plasma membrane. The Rh1 protein of the green alga *C. reinhardtii* was proposed to function as a CO_2_ channel [34, 89], and human Rh30/RhAG proteins to contribute to the CO_2_ permeability in RBCs [90–92]. While the evidence was merely transcriptional, hence indirect, for Rh1 from *C. reinhardtii* and conflicting data have been obtained for human Rh proteins, the identification of a potential CO_2_-binding pocket in *Ne*Rh50 crystal structure supported the idea that rhesus proteins might act as gas channels for CO_2_ in addition to NH_3_ [84]. At the same time*, in vivo* characterization of *Ne*Rh50 showed no evidence of a CO_2_-dependent growth effect in a knockout mutant [88]. Another argument against Rh involvement in CO_2_ was presented in a later report monitoring transmembrane CO_2_ flux, which concluded that protein-facilitated transport of CO_2_ is highly improbable [93]. In addition, a CO_2_-binding pocket was not detected in the crystal structure of human RhCG, which was proposed to be very representative of all Rh homologues [60]. Finally, a study showed that during long-equilibration molecular dynamics (MD) simulations CO_2_ molecules do not show any tendency to diffuse across the periplasmic vestibule of either bacterial or human Rh50 proteins [94].

**Table 2.** Summary of transport mechanism in bacterial ammonium transporters after 2004

Organism	Protein	System	Method	Mechanism	References
* A. fulgidus *	Amt-1	Proteoliposomes	EP	Electrogenic transport	[98]
	Amt-3	Proteoliposomes	EP	Electrogenic transport	[98]
* K. stuttgartiensis *	Amt-5	Proteoliposomes	EP	NH_4_^+^ binding only	[70]
* E. coli *	AmtB	Proteoliposomes	pH	NH_3_ transport	[65]
		* E. coli* cells	TA	Channel-like transport	[80]
		Proteoliposomes	EP	Electrogenic transport	[67, 76]
* N. europaea *	Rh50	Washed assay	TA	NH_3_ transport	[87]
		Proteoliposomes	EP	Electrogenic transport	[76]

EP, electrophysiological measurements; TA, ^14^[C]-methylammonium transport assay; pH, pH measurement.

## Finally; the activity of bacterial Amt and Rh proteins is electrogenic!

The view that Amt/Mep/Rh proteins conducted a neutral species was first experimentally challenged for some plant Amt and Rh proteins [95, 96], with charge translocation observed for both AMT1;1 from *Lycopersicon esculentum* and human RhAG expressed in *Xenopus laevis* oocytes [53, 95]. Concerning bacteria, in 2011, using a systems biological approach, Boogerd *et al.* provided evidence that AmtB-mediated import must be active for intracellular NH_4_^+^ concentrations to sustain bacterial growth [97]. The first direct evidence for electrogenic transport of ammonium in bacteria was proposed after *in vitro* analysis of Amt1 and Amt3 from *A. fulgidus* reconstituted in artificial liposomes using SSME (Table 1) [98]. Afterward, the activity of AmtB and *Ne*Rh50 was also found to be electrogenic using the same electrophysiological approach (Table 1 [76, 99].

The apparent conflict between the crystal structures, in which certain structural features suggest that an electrogenic transport would be disfavoured, and functional observations of electrogenic transport reignited the controversies regarding the transport mechanism of Amt/Rh, centring on a new paradox: how can a charge travel through a hydrophobic pore?

Before answering this question, it is important to explore the different mechanisms that may explain the electrogenicity of transport. Three potential mechanisms are possible: NH_4_^+^ uniport, NH_4_^+^/H^+^ symport, NH_4_^+^ deprotonation and subsequent symport of NH_3_ and H^+^. Of these, the translocation of NH_4_^+^ is the least likely, due to the energetic barriers preventing ion conduction through a hydrophobic pore. Additionally, it has been shown that ‘open-pore’ mutants of AmtB (constructed with the Phe-gate removed) become inactive in transporting MeA and do not gain permeability to either H^+^ or K^+^ [73]. This demonstrates that widening access to the pore does not allow for ion conduction through AmtB and suggests that another mechanism and/or residues must be implicated in transport.

### Mechanisms of deprotonation

A breakthrough came from a study that experimentally demonstrated the importance of NH_4_^+^ deprotonation in ammonium transport by some Mep and Amt proteins [100]. Ariz *et al.* used the natural chemical/physical properties of the N-isotopic signature linked to NH_4_^+^/NH_3_ interconversion, and showed that only *S. cerevisiae* cells expressing some Amt/Mep proteins were depleted in ^15^N relative to ^14^N when compared to the external ammonium source [100]. They showed that this isotope fractionation can only be explained by the deprotonation of NH_4_^+^ before the translocation of NH_3_ by Amt/Mep proteins. However, the mechanism of deprotonation is still a matter of debate.

#### Deprotonation at the S1 site

Three mechanisms have been proposed for NH_4_^+^ deprotonation at the S1-binding site. The first hypothesis was proposed by Khademi *et al.* [65]. They found that when bound to the S1 site, the pKa of NH_4_^+^ decreased below 6, which favours the deprotonation of a NH_4_^+^ by a water molecule. They also measured a NH_3_ flux through the pore of AmtB using a fluorescence-based assay to measure the pH variation in proteoliposomes containing AmtB after an ammonium pulse. Initially in agreement with this hypothesis, Fritz Winkler argued that the deprotonation happens before reaching the hydrophobic pore, based on a Michaelis–Menten kinetics model, and deduced that the most likely deprotonation occurs at the S1-binding site via water molecules, thus the proton should be released in the periplasm [10]. Further computational simulations substantiate this hypothesis: combining the potential of mean force with data from thermodynamic integration to calculate the apparent pKa of NH_4_^+^/NH_3_ along the translocation pathway, it was deduced that the most likely proton acceptor was a water molecule around the S1 site [101, 102], releasing the hydronium ions back in the bulk solution. However, it has now clearly been demonstrated that Amt/Rh-mediated transport in bacteria is electrogenic, which contradicts this potential mechanism of deprotonation [67, 76, 98].

Secondly, computational modelling suggested that at the S1 site, S219 fulfils a dual role in ammonium transport: firstly coordinating NH_4_^+^ via H-bond formation between the hydroxyl oxygen of S219 before acting as a proton acceptor to facilitate deprotonation [103]. This model has remained relatively untested – but Javelle *et al.* showed that AmtB^S219A^ had enhanced, rather than reduced, MeA and ammonium uptake compared to the WT when tested using MeA transport assays or SSME, respectively [73]. These results suggest that under the *in vivo* and *in vitro* conditions used in these studies, recruitment at S1 was not rate limiting and that the hydroxyl moiety of the S219 residue is unlikely to be involved in the deprotonation mechanism.

The third possibility for deprotonation at the S1 site is that the carboxyl moiety of the D160 residue acts as the proton acceptor. Multiple sequence alignments of Amt/Mep/Rh proteins show that D160 is highly conserved, potentially indicating an important functional role [59, 74, 104]. It was demonstrated by *in vivo* MeA uptake that the AmtB^D160A^ variant was unable to transport ammonium, whereas AmtB^D160E^ was capable of maintaining 70 % of the WT activity, suggesting that the D160 residue is essential for AmtB activity [74]. However, it is important to note that this study was conducted *in vivo* using MeA as a substrate, and there are numerous examples where the activity of AmtB variants measured *in vivo* using MeA as a substrate does not mirror the activity measured in *in vitro* systems using ammonium [76, 77, 105], for example AmtB^D160E^ (see below). This shows that MeA is a poor substrate analogue. In contrast, the AmtB X-ray structure revealed later that the distance between the D160 residue and the binding site is not favourable for a direct interaction with the substrate [65, 66]. Further computational studies, using umbrella sampling simulations, indicate that the residue D160 is important for the structural stability of the periplasmic vestibule but not involved in the mechanism of ammonium transduction [106]. However, this contradicted MD simulation studies previously performed by Luzhkov and co-workers showing that the D160 residue, separated by 8 Å from the binding site, was able to interact with ammonium [107]. A potential indirect role of D160 on NH_4_^+^ deprotonation has also been proposed: a hydrogen bond wire between NH_4_^+^ at the S1 site and the carboxylate group of D160 via two water molecules was observed using MD simulation. Thus, the authors concluded that D160 is most likely the proton acceptor from NH_4_^+^ [108, 109]. More recently, combining SSME analysis, yeast complementation assay and advanced MD simulation revealed that AmtB^D160A^ and AmtB^D160E^ variants are transport-deficient, suggesting that residue D160 plays a central role in the transport mechanism [76]. Moreover, the fact that the conservative D to E substitution at position 160 impairs ammonium transport via AmtB indicates that D160 not only participates in electrostatic interactions with NH_4_^+^ at the S1 site, as previously hypothesized [102, 107], but is also involved in the translocation mechanism. Furthermore, residue D160 was proven to contribute to stabilizing the water wire that bridges the S1 site to the twin-His motif in the hydrophobic pore (see below and Fig. 2). Thus, while D160 is clearly important, the extent of its role in the transport mechanism and, more specifically, in NH_4_^+^ deprotonation is still a matter of debate.

#### Deprotonation at the Phe-gate

In 2008, it was noted that the F107A mutation has no effect on AmtB activity [73]. However, an AmtB^F215A^ variant could not transport ammonium or MeA. Similarly, replacing the F215 with H, E, S or W residues also abolished transport activity. This result demonstrated the importance of the F215 residue and it was speculated that the deprotonation of ammonium may occur during the passage through the Phe-gate [73]. This hypothesis was further substantiated by computational calculation; the apparent pKa for NH_4_^+^ changes from 4 to −16 pKa units (ΔpKa compared to normal NH_4_^+^/NH_3_ pKa at the S1 and S2 sites) when moving from the S1 to the S2 site, which indicates that ammonium may be deprotonated only after entering the constricted part of the channel pore, near the Phe-gate position [107]. However, no evidence of the direct role of the residue F215 in the deprotonation has been obtained so far.

#### Deprotonation at the S2 position

A 2006 MD study indicated that the carbonyl group of the A162 residue can be orientated toward either the S1 or the S2 site (Fig. 1), forming an H-bond with NH_4_^+^ [110]. This result suggested that the A162 residue acted as a ‘substrate guide’ from the binding site across the Phe-gate to the S2 site. A 180 degree flip of the Phe-gate was frequently observed during these MD simulations; hence it was assumed that the deprotonation occurred after the S1 site and near to the S2 site. The deprotonation mechanism deduced by Nygaard and co-workers proposed that NH_4_^+^ is translocated between the S1 and S2 site by the movement of A162, which became perfectly aligned with the G163 : NH bond by interacting with the O_δ_ of the D160 residue. This new amino acid configuration allowed the deprotonation of NH_4_^+^ by A162 and its transfer to D160. Once a proton has been transferred from NH_4_^+^ to the O_δ_ of the D160 residue, all amino acids involved become neutral and the C-O-H group of A162 is reorientated to the S1-binding site and the proton released in the periplasmic vestibule via a water molecule [110]. However, as already pointed out above, this would imply an electroneutral activity for ammonium translocation, which is not the case [76, 98]

Lamoureux and co-workers, using MD simulations of AmtB, have shown that a wire composed of three water molecules was present along the hydrophobic pore, corresponding to the position (S2–S4) of the residual electron densities observed in the original structure published previously [65, 111]. By using protonation states and potential mean force calculations, the authors suggested that the deprotonation occurs at the S2 site. Two mechanisms have been proposed: firstly, a deprotonation via the H168 residue of the twin-His motif, and transfer of the proton in the lumen via the twin-His motif, or secondly, a deprotonation by the water molecule present in the vicinity of the S2 site and transfer of the proton in the lumen through the water wire via a Grotthuss mechanism (Decoursey, 2003) [112, 113]. However, a study combining MD with functional assays (using a growth experiment or MeA uptake assay) was carried out by Mike Merrick’s group, which challenged the deprotonation hypothesis via the H168 residue [105]. Numerous variants of the twin-His motif of AmtB and Mep2 from *S. cerevisae* were analysed and it was shown that single His mutants (AmtB^H168A or H318A^ and Mep2^H194A or H348A^) were still capable of growth on minimal medium containing 1 mM of ammonium as the sole nitrogen source, but were unable to translocate MeA inside the cell [105]. The apparent discrepancy between both *in vivo* assays (growth analysis and MeA uptake) was explained by MD simulations, which revealed that the single histidine H168A variant allowed ammonia to pass across the pore but not MeA [105]. Recently, the analysis of a series of variants in which alanine residues were introduced alone or in combination in the twin-His motif showed that AmtB switches from transporter to channel-like activity in the absence of the twin-His motif, directly translocating hydrated NH_4_^+^ through the pore [76]. Taken together, these results suggest that the H168 residue is not essential for NH_4_^+^ deprotonation, but further analysis is still needed to understand the exact role of the twin-His motif in Amt/Mep/Rh activities.

### How does a charge travel through a hydrophobic pore?

**Fig. 4.**
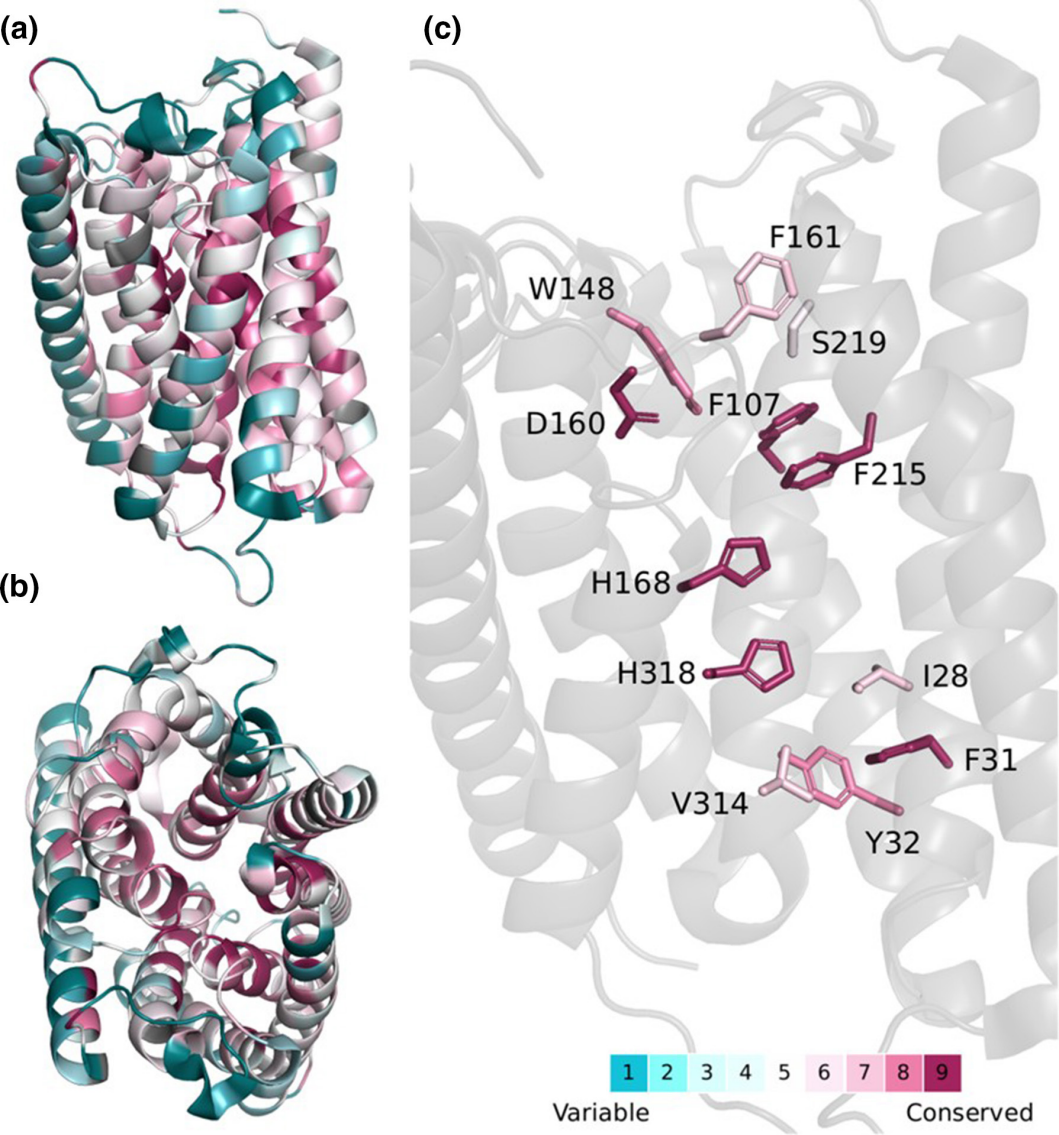
Evolutionary conservation of the proton and hydrophobic pathways for H^+^ and NH_3_ translocation in AmtB. Evolutionary conservation scores indicate that the residues that line both the water wires and the internal hydrophobic pathway show a large degree of conservation among homologous proteins. Evolutionary conservation scores were estimated using the ConSurf server [119]. In brief, a multiple sequence alignment (MSA) was built of homologues collected from the SWISS-PROT database. Homologues were identified by five iterations of HMMER, producing an MSA of 64 sequences. The evolutionary conservation scores are projected onto an AmtB monomer (PDB code: 1U7G), with some helices removed for clarity. (**a**) Side view. (**b**) Top view from periplasmic side. (**c**) Close-up view of key residues involved in the two-lane mechanism. Reproduced from [76].

As we have seen, although the mechanism of deprotonation remains elusive, there is increasing evidence that suggests deprotonation and co-transport of NH_3_/H^+^ as a likely mechanism for ammonium translocation, although the route the proton takes to get into the cytoplasm remains to be determined. Based on MD simulations, it was proposed that the pathway for protons, following NH_4_^+^ deprotonation, involves translocation via a Grotthuss mechanism through water chains formed in the pore or passage through the ‘twin-His’ motif [111]. A recent study combining *in silico*, *in vitro* and *in vivo* approaches, offers an attractive model and experimental evidence explaining the fate of the proton following NH_4_^+^ deprotonation [76]. Through extended MD simulation, two interconnected water wires (WWs), bridged by the twin-His motif, connecting the periplasmic and cytoplasmic regions of the protein, were identified in AmtB. These results led to the hypothesis that the WWs allow for a continuous pathway for proton transfer from the S1 NH_4_^+^ sequestration region to the cytoplasm. Using SSME assays and the reduced deuteron mobility of heavy water (D_2_O), it has been experimentally proven that WWs are indeed essential for proton conduction during the AmtB transport cycle [76]. Collectively, these results indicate that in AmtB NH_4_^+^ undergoes deprotonation, allowing the passage of uncharged NH_3_ molecules through the central part of the channel lined by the hydrophobic groups into the cytoplasm, while H^+^ ions are translocated a via parallel pathway outlined by the WWs. The passage of NH_3_ and H^+^ is followed by subsequent reprotonation in the cytoplasm driven by intracellular pH (Fig. 2) [76]. This mechanism defines a new principle of achieving transport selectivity against competing ions in a biological transport process. In the same study, Williamson *et al.* provided evidence that the activity of the *N. europaea* Rh50 ammonium transporter is in fact electrogenic (Table 1), as opposed to the previously proposed NH_3_ channel [76, 87, 88]. While the sequence similarity within the Amt/Rh family is low, the segments involved in the proposed transport mechanism are highly conserved (Fig. 4); hence, the authors proposed that this mechanism could be shared between various members of the Amt/Mep/Rh family [76].

## Conclusion and perspectives: is there a common mechanism in the Amt/Mep/Rh protein family?

It is now clear that the activity of bacterial ammonium transporters studied so far is electrogenic. An original model explaining how a charge can be translocated via the hydrophobic pore has been proposed [76]. However, whether this is a conserved mechanism of transport amongst the Amt/Mep/Rh family remains an open question. Reviewing the mechanistic information to date for non-bacterial Amt/Mep/Rh and its implications is a huge undertaking and is beyond the scope of this review, although some insight can be drawn from the work to date.

Firstly, the residues that delineate and stabilize the WWs (Fig. 2) in AmtB are highly conserved across the whole Amt/Mep/Rh family (Fig. 4). This indicates that the model proposed by Williamson *et al.* may be conserved amongst all members of the family that exhibit electrogenic activity [76]; however, it is clear that some Amt, Mep and Rh proteins may exhibit an electroneutral activity.

The transceptor function of Mep2 in the filamentation signalling in *S. cerevisiae* may hint at a different mechanism of transport [77, 114]. Currently, it is unknown how the signal that leads to the pseudohyphal growth is initiated, but two possibilities are currently envisaged: Mep2 binds to a partner protein to illicit the signal, or Mep2 conducts ammonium via a separate mechanism to Mep1/3 to transduce the signal. It has been shown recently using electrophysiology in *Xenopus* oocytes expressing either *S. cerevisiae* Mep1 or Mep2 transporter that their activity is electrogenic and electroneutral, respectively [114]. In addition, the first experiments with purified Mep2 reconstituted into artificial liposomes produced no observable current using SSME measurement, also suggesting an electroneutral transport mechanism (Boeckstaens *et al*., unpublished results). The molecular basis of the difference between electrogenic Mep1 and electroneutral Mep2 remains, however, completely unknown.

Plant Amts are also the subject of much research. In plants, the mechanism of ammonium transport is best understood in *A. thaliana*, where the genome encodes six AMT genes, divided into two sub-families, AMT1 and AMT2. The AMT1 sub-family have been demonstrated to facilitate electrogenic transport in *A. thaliana* and in other plants, although it has been shown that the AMT2 family activity is electroneutral. For a review, see Ludewig *et al.* and Hao *et al.* and references therein [115, 116].

As the most distant member of the Amt/Mep/Rh family, distinct functions have repeatedly been hypothesized for the Rh proteins. A subset of the family (Rh30) has evolved to lose transport activity and instead act as antigens on the surface of erythrocytes. Rh50 proteins are known to be transporters, but their substrate and mechanism remain debated. The Rh50 protein from *N. europaea* and the human RhAG and RhCG complement Δ*mep1-3 S. cerevisae* [27, 88], suggesting that they also transport ammonium. Attempts to elucidate the mechanism, however, have been conflicting. Initial patch-clamp physiology experiments wherein RhAG expressed in *Xenopus* oocytes led the authors to propose that RhAG is an NH_4_^+^/H^+^ antiporter [58]. Contemporaneous studies reported that RhAG and RhBG were electroneutral, and RhCG was an electrogenic NH_4_^+^ uniporter [53]. However, later work using stopped-flow spectrophotometry to measure changes in pH following an ammonium pulse in erythrocyte vesicles from human and mouse genetic variants and liposomes containing RhCG concluded that RhAG and RhCG were both NH_3_ channels [117, 118]. This is supported by more recent oocyte experiments, wherein no significant difference in current was observed in Rhcg-expressing oocytes compared to control [95]. Despite this lack of current, Rhcg resulted in increased surface acidification, leading the authors to conclude that Rhcg transported NH_3_ but not NH_4_^+^ [95].

Hence, whether there is a conserved, or partially conserved, mechanism of transport amongst the Amt/Mep/Rh family remains open. Further, important questions remain open concerning the site of deprotonation, which seems to be a common mechanism of all Amt/Mep/Rh studied so far, and residues involved in the process. The energetics of the transport and the dynamics of the protein during the transport cycle also remain open questions. Hopefully, the next three decades of research will bring answers to these questions, improving our understanding of the family of these proteins.
